# Lifestyle modification practice and associated factors among diagnosed hypertensive patients in selected hospitals, South Ethiopia

**DOI:** 10.1186/s40885-017-0081-1

**Published:** 2017-12-04

**Authors:** Eyasu Siyum Buda, Lolemo Kelbiso Hanfore, Robera Olana Fite, Alula Seyum Buda

**Affiliations:** 1 Doyogena Woreda Health Office, Medical Services Core Process Coordinator, SNNPR, Doyogena, Ethiopia; 2 College of Health Sciences and Medicine, Department of Nursing, Wolaita Sodo University, PO Box 138, Wolaita Sodo, Ethiopia; 3 Faculty of Health Sciences, Department of Nursing, Wachemo University, Hosaena, Ethiopia

**Keywords:** Hypertension, Lifestyle modification, Practice, Hospital, Durame, SNNPR

## Abstract

**Background:**

Hypertension is one of the leading causes of disability and death in both developed and developing countries that need urgent strategies to implement interventions that control it. Appropriate lifestyle changes often called non-pharmacological approaches that often overlooked are the corner stone of the prevention and control of hypertension. The aim of this study is to assess the practice of lifestyle modifications and associated factors among diagnosed hypertensive patients in Durame and Nigist Elleni Mohamed Memorial General Hospitals in southern Ethiopia.

**Methods:**

Facility-based cross-sectional study was conducted among 205 hypertensive patients in Durame and Nigist Elleni Mohamed Memorial General Hospitals in Sothern Nation and Nationality People Representative (SNNPR), from March 1-30 2016. Simple random sampling was used to select study subjects. Data were entered to Epidata 3.1 and exported to Statistical Package for Social Sciences (SPSS) version 20.0 for analysis. A binary Logistic regression model was fitted to determine independent predictors of lifestyle modifications among hypertensive patients. Adjusted odds ratio at 95%CI was used to declaring the independent effect of each variable on the outcome variable.

**Result:**

The study revealed that only 56(27.3%) of the patients practiced recommended lifestyle modifications. The study found that age (Adjusted Odds Ratio [AOR] = 0.27, 95% Confidence Interval [CI]:0.13-0.61), educational status (AOR = 2.00,95% CI:1.33-6.75), monthly income (AOR = 2.46, 95% CI:1.32-4.63), years since diagnosis (AOR = 2.48, 95%CI: 1.32-4.69), and co-morbidity (AOR = 0.28,95% CI: 0.13-0.61) were factors significantly associated with lifestyle modification practice (*p* < 0.05).

**Conclusion:**

Generally, lifestyle modification practices among hypertensive patients were low in this study. Therefore, Patients should be educated on the recommended lifestyle modifications that may help patients to control f their blood pressure.

## Background

Hypertension is a major health problem in developed countries and now becoming an increasing important cause of morbidity and mortality in developing countries. Today one in three adults has hypertension. Hypertension is a global public health challenge due to its high prevalence and the associated risk of stroke and cardiovascular diseases in adults. It is estimated to cause 7.5 million deaths worldwide and about 12.8% of the total annual deaths in SSA [1–3].

The risk factors associated with hypertension are tobacco use, alcohol consumption, obesity, high cholesterol and diabetes mellitus. Tobacco use increases the risk of complications among those with hypertension. In 2008, one billion people were smokers and the global prevalence of obesity has nearly doubled since 1980 [4, 5].

Disease burden is closely related to the average volume of alcohol consumption, and, for every unit of exposure, is strongest in poor people and in those who are marginalized from society [6, 7]. In 2012, about 3.3 million deaths, or 5.9% of all global deaths, were attributable to alcohol consumption [8]. If inactivity were decreased by 10% more than 533,000 deaths can be averted. Furthermore, if inactivity is decreased by 25%, more than 1.3 million deaths can be prevented [9, 10]. Body mass index (BMI) is positively and independently associated with morbidity and mortality from hypertension [11, 12].

Lifestyle modification is the foundation of preventive management in individuals with hypertension. It is recommended as an initial treatment before the start of drug therapy and as an adjunct to medication in those already on drug therapy. Lifestyle modification may facilitate drug step-down and drug withdrawal in highly motivated individuals who achieve and maintain lifestyle changes [13–16]. A lifestyle modification includes weight reduction, salt restriction, and physical activity, smoking cessation and abstaining from alcohol [17, 18].

The World Health Organization (WHO) reported that hypertension is responsible for 62% of cases of cerebrovascular disease and 49% of cases of ischemic heart disease. In addition, hypertension is the topmost risk factor for death worldwide [13, 19]. In different studies age, marital status, income, the source from which they get information, the existence of co-morbidity, sex differences and individual’s knowledge on hypertension are factors found to influence lifestyle modification practice [20–24].

The balance of energy intake and exercise is an important determinant of hypertension [25]. As suggested WHO, the normal weight for an adult over 18 years is less than or equal to 18.5–24.9. BMI that is greater than this puts one at risk of obesity-related diseases as high blood pressure [26].

Physical Inactivity causes 9% of premature mortality or more than 5.3 million of the 57 million deaths that occurred worldwide in 2008. If inactivity were not eliminated but decreased instead by 10 or 25%, more than 533, 000 and more than 1·3 million deaths, respectively, could be averted every year [9, 10].

In Africa, as elsewhere, obesity and sodium intake are risk factors for hypertension. In industrialized societies such as the United States, obesity accounts for 25% of cases of hypertension. However, due to the relative leanness of Africans, the contribution of obesity to high blood pressure is only around 10% [27].

In spite of emerging empirical evidence of the efficacy of lifestyle modification in blood pressure control, little is known about the practice of lifestyle modification and associated factors among hypertensive patients in Ethiopia. The study considers that practice of lifestyles and associated factors and their relationships will guide to facilitating for actions for greater practice among the hypertensive patients. Therefore, finding from the study would alert health professionals, government and other stakeholders on lifestyles and factors associated with the practice of these lifestyles to control hypertension.

## Methods

### Study area and period

The study was conducted in Durame and hosanna towns. Durame is the administrative town of Kembata Tembaro Zone which is found SNNPR and located 285 km from Addis Ababa and 125 km from Hawassa which is the capital city of the region. The zone has seven Weredas and three town administration with the estimated population of 857,084. The durame hospital is the general hospital found in the zone. Hosanna town is also the administrative town for Hadiya zone that is 232kms from the capital Addis Ababa and 194 km west of Hawassa. The zone has estimated population of 1,506,733. Nigist Ellen Mohammed memorial General Hospital is a governmental hospital which is found in the Hosanna town. The study was conducted from March 1to April 30, 2016.

### Study design

The facility-based cross-sectional study was conducted in Durame and Nigist Elleni Mohamed memorial general hospitals.

### Population

The source population was all hypertensive patients who were treated in Durame general hospital and Nigist Ellen Mohammed memorial general hospitals. The study population was randomly selected hypertensive patients who came for follow up during the study period.

### Inclusion and exclusion criteria

All hypertensive patients’ age ≥ 18 years were included in the study. Patients who were severely ill and not able to communicate were excluded from the study.

### Sample size determination

The sample size was determined using a single population proportion by assuming that 50% proportion of the patients practiced lifestyle modifications with 95% confidence interval and 5% margin of error. Using population correction formula and adding 10% non-response rate the sample size was 210.

### Sampling technique

Total adult hypertensive follow-up patients are 165 in Durame and 210 in Nigist Ellen Mohammed memorial hospital. Therefore, the total patients registered for follow up in both hospitals were 375. The sample size was allocated to both hospitals proportionally. All previously, registered 375 patients were included in the sampling frame. Then the study respondents were selected using random sampling technique. The list of patients (sampling frame) was obtained from the registration books of the patients registered for follow up in hospitals and study subjects were selected by lottery method.

### Variables of the study

#### Dependent variable

The practice of lifestyles modifications.

#### Independent variables

**Socio-Economic variables;** Age, sex, income, marital status, educational status, religion, occupation, ethnicity, residence.

**Health profile of the patients;** Duration of diagnosis, presence of co-morbidity, family history of hypertension.

**Source of information about lifestyles; −** medical personnel, media, friends, and family.

**Individual factors**; knowledge of hypertension.

### Data collection instrument

The questionnaire has socio-Economic, questions related with the source of information about lifestyles, knowledge on hypertension and lifestyles, questions related to lifestyle modification practices and questions about health profile of the patients. The lifestyle modification practices were measured using questionnaires adapted from hypertension self-care practice questions which are recommended by joint national committee on detection, prevention evaluation and treatment of hypertension (JNC7) and WHO STEPS questionnaires [18, 28].

### Measurements

**Low-salt diet -** ten items were used to assess practices related to eating a healthy diet, avoiding salt while cooking and eating, and avoiding foods high in salt content. A mean score was calculated. Scores of five or better indicate that patients followed the low-salt diet and considered as having good low salt diet practice.

**Physical activity -** Physical activity was assessed by two items. “How many of the past 7 days did you do at least 30 minutes total of physical activity?” and “how many of the past 7 days did you do a specific exercise activity (such as swimming, walking, or biking) other than what you do around the house or as part of your work?” Responses were summed (Range 0–14) patients who scored eight and above were coded as a having good physical activity practice. All others coded as poor practice.

**Smoking -** Smoking status was assessed with one item, “How many of the past 7 days did you smoke a cigarette?” Respondents who reported 0 days were considered a nonsmoker.

**Weight management -** ten items assessed using activities undertaken to manage weight through dietary practices such as reducing portion size and making food substitutions as well as exercising to lose weight. Items assessed agreement with weight management activities during the past 30 days. Response categories ranged from strongly disagree (1) to strongly agree (5). Responses were summed creating a range of scores from 10 to 50. Participants who report that they agreed or strongly agreed with all ten items (score ≥ 40) were considered to have good weight management practice.

**Alcohol -** Alcohol intake was assessed using 3-item Participants who report not drinking any alcohol in the last 7 days or who indicated that they usually did not drink at all were considered abstainers. All others were considered as not having a good practice of alcohol consumption.

Height was measured using portable stadiometer without participant wearing shoes to the nearest 0.5 cm. reading was taken after the participant was requested to have feet together heals against the back board, knees straight and look straight forward. In addition, weight, to the nearest 0.1 kg. Body mass index (BMI) was calculated from the weight and height. BMI (kg/m2) was categorized as normal weight (18.5 ≤ BMI < 24), overweight (24 ≤ BMI < 28), and obese (BMI ≥ 28) using the using WHO recommendations [28].

### Data collection procedures

Data were collected by two trained diploma nurses and using face to face interview method. One BSc nurse supervisor was assigned to each hospital. The socio-demographic, health profiles of participants, knowledge on hypertension, and source of information of the study participants were collected using an interview based structured questionnaire adapted from the WHO manual and reviewing different literatures [28]. Physical characteristics (height and weight) were measured. The lifestyles practices were measured using a tool adapted to the local context from hypertension self-care activity scales [18].

Lifestyle modification practice was measured using physical exercise, low salt diet, alcohol consumption, smoking and weight management practices. The lifestyle modification practice was classified as a ‘good practice’ and ‘poor practice’. Respondents were labeled to have “good” ‘lifestyle modification practices if they scored above the mean in all recommended lifestyle questions. Weight and height of the patients were measured and BMI was calculated and classified using WHO guideline as normal weight, overweight and obese. Weight and height measurements were taken during data collection.

### Data quality control

The questionnaire was prepared in English then translated into Amharic language and was back translated into the English language by another person to check its consistency.

The questionnaire was pre-tested in 5% of total eligible patients in Butajira hospital for their accuracy and consistency prior to actual data collection. Furthermore, the supervisor and the investigator were given feedback and corrections on daily basis to the data collectors. Completion, accuracy, and clarity of the collected data were checked carefully on a regular basis. The data was carefully entered and cleaned before the beginning of the analysis.

### Data processing and analysis

After collection, data were checked for completeness and were entered into Epidata 3.1 version and exported to SPSS 20.0 version for further analysis. Descriptive statistical analysis such as Proportion, frequency distribution, means, and the measure of dispersion was used to describe data and analytical statistics including bivariate and multivariable logistic regression analysis was done. Bivariate logistic regression was done to examine the association between dependent and independent variables. After running bivariate logistic regressions, all variables with *p* < 0.25 was considered as a candidate for the final model and corresponding *p*-value of <0.05 was considered as statistically significant. Adjusted odds ratio at 95% CI was considered to declare the independent effect of independents variables on the outcome. Finally, results were presented using charts and tables.

### Operational definitions

**Good lifestyle modification Practice:** when patients respond the mean or above the mean score on practice questions.

**Poor lifestyle modification practice:** when patients respond below the mean score on practice questions.

## Result

### Socio-demographic characteristics of study subjects

Out of 210 study participants, 205(97.5%) participated in the study. More than half 105(51.2%) of them were females. The mean age of respondents was 53.9(SD ± 9.64 years). About two-third 136(66.3%) of participants were married. Regarding the educational status of subjects, 118(42.4%) has no formal education. Concerning their employment status, 63(30.7%) were government employees. 105(51.7%) were Protestants in their religion and 102(41%) of the study, subjects were Hadiya in Ethnicity. Average monthly income for 48(23.4%) of them was less than 500 ETB (Table 1).

**Table 1:** Socio-demographic characteristics of hypertensive patients in Durame and Nigist Elleni Mohamed Memorial General Hospitals, SNNPR, May 2016 (N = 205)

Variables	Frequency	Percent (%)
Age		
< 65 years	174	84.9
> =65 years	12	15.1
Sex		
Male	100	48.8
Female	105	51.2
Marital status		
Married	136	66.3
Single	24	11.7
Divorced	15	7.3
Widowed	30	14.6
Educational status		
Formal education	87	57.6
No formal education	118	42.4
Employment status		
Government employee	63	30.7
Private employee	63	30.7
Merchant	36	17.6
Housewife	23	11.2
Daily laborer	14	6.8
Retired	6	2.9
Religion		
Orthodox	65	31.7
Protestant	105	51.2
Muslim	10	4.9
Catholic	25	12.2
Ethnicity		
Hadiya	102	49.8
Kembata	57	27.8
Gurage	21	10.2
Wolaita	16	7.8
others	9	4.4
Average monthly Income		
< 500ETB	48	23.4
500-999 ETB	61	29.8
> =1000 ETB	96	46.8

### Lifestyle modification practice

**Fig. 1:**
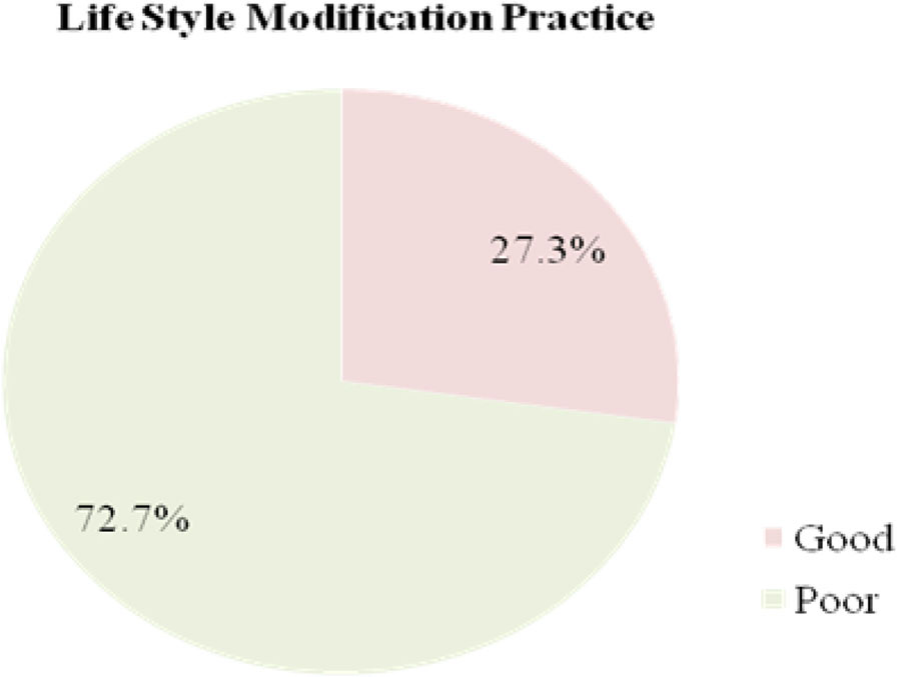
Life style modification practice among hypertensive patients in Durame and Nigist Elleni Mohamed Memorial General Hospitals, SNNP, 2016(*N* = 205)

The mean score for lifestyle modification practice was 40(SD ± 14.36). Only 56(27.3%) patients practiced recommended lifestyle modification. The mean (+SD) score for physical activity was 4.46(±3.45) with the maximum score of 14. Only 33 (16.1%) practiced physical activity for 30 min per day. The mean (+SD) score of weight management practice of the patients were 29.36(±13.43) with the maximum score of 50. Eighty-six (41.9%) had good weight management practice. The mean (±SD) score for low salt diet was 4.59(±2.03). From the patients, 118 (57.5%) practiced recommended low diet salt. One hundred eighty (87.9%) did not drink alcohol in the last 7 days. One hundred eighty-seven (91.2%) were not a smoker (Fig. 1 and Table 2).

**Table 2:** Life style modification practices among hypertensive patients in Durame and Nigist Elleni Mohamed Memorial General Hospitals, SNNPR, May 2016 (N = 205)

Variables	Frequency	Percent
Practice physical activity for thirty minutes per day (*N* = 205)		
Yes	33	16.1
No	172	73.9
Weight management practice (*N* = 147)		
Good	86	41.9
Bad	61	58.1
Practice recommended low salt diet (*N* = 205)		
Yes	118	57.5
No	87	42.5
Alcohol consumption (*N* = 205)		
Yes	180	87.9
No	25	12.1
Smoking status (*N* = 205)		
Yes	18	10.8
No	187	91.2

### Health profile related, individual related and source of information related factors

From the patients who had basic knowledge about hypertension 23 (25%) practiced good lifestyle modification and those who were in treatment for 5 to 10 years (13.6%) practiced good lifestyle modification. From those who had a family history of hypertension 20(31.7%) practiced good lifestyle modification. From those who were informed by health professionals 31(35.2%) practiced good lifestyle modification. Twenty three (25.8%) of patients with co-morbidity practiced good lifestyle modification (Table 3).

**Table 3:** Health profile related, individual related and source of information related factors among hypertensive patients in Durame and Nigist Elleni Mohamed Memorial General Hospitals, SNNP, 2016(N = 205)

Variables	Lifestyle Modification Practice	
	Good	Poor
	N (%)	N (%)
Knowledgeable about Hypertension		
Yes	23(25.0)	68(75)
No	33(29.2)	80(70.8)
Duration since diagnosis		
< 2 years	6(13.6)	38(86.4)
2-5 years	30(33.3)	60(66.7)
5-10 years	19(28.4)	48(71.6)
> 10 years	1(25.0)	3(75.0)
Family history of Hypertension		
Yes	20(31.7)	43(68.3)
No	36(25.4)	106(74.6)
Hear information about lifestyles		
Yes	39(33.1)	79(66.9)
No	17(19.5)	70(80.5)
Source of information		
Health professionals	31(35.2)	57(64.8)
Different medias	6(33.3)	12(66.7)
Family and friends	3(21.4)	11(78.6)
Co-morbidity		
Yes	23(25.8)	66(74.2)
No	33(28.4)	83(71.6)

### Factors associated with lifestyle modification practice among hypertensive patients

Age, Marital status, educational status, monthly income, duration of diagnosis, had information about lifestyle, the source of information, co-morbidity status were entered into the final model. According to the result of the multivariable analysis, Age, duration since diagnosis, average monthly income, educational status, and co-morbidity were independent predictors of good lifestyle modification practice among hypertensive patients.

Patients aged greater than 65 years were 72% less likely to have good lifestyle modification practice (AOR = 0.28, 95% CI: 0.13-0.61) than patients with below 65 years. On the other hand, hypertensive patients with income of 1000 ETB were 2.4 times more likely to practice good lifestyle modification (AOR = 2.38, 95% CI:1.15-5.57) as compared to patients with income of less than 500ETB.

Patients without formal education were 2 times more likely practice good lifestyle modification (AOR = 2.00, 95% CI: 1.33-6.75) as compared to those who had formal education.

Individuals with five to 10 years treatment duration were 2.5 times more likely to practice lifestyle modification (AOR = 2.48, 95% CI: 1.32-4.69) as compared to those on treatment for less 2 years treatment. On the other hand, patients who were with co-morbidity were 72% less likely to practice good lifestyle modification (AOR = 0.28, 95% CI: 0.13-0.61) as compared to those without co-morbidity (Table 4).

**Table 4:** Predicators of lifestyle modification practice among hypertensive patients in Durame and Nigist Elleni Mohamed Memorial General Hospitals, SNNP, 2016(N = 205)

Variables	Lifestyle modification practice		COR (95%CI)	AOR (95%CI)
	Good	Poor		
	N (%)	N (%)		
Age				
< 65 yrs	45(25.9)	129(74.1)		1
> = 65 yrs	11(5.4)	20(9.8)	1.58(0.70-3.54)	0.27(0.13-0.61)*
Marital status				
Married	34(25.0)	102(75.0)		1
Single	10(41.7)	14(58.3)	2.14(0.45-2.68)	1.08(0.45-2.58)
Divorced	4(26.7)	11(73.3)	0.92(0.27-3.06)	1.09(0.22-1.78)
Widowed	8(26.7)	22(73.3)	1.09(1.04-4.06)	1.33(0.60-2.94)
Educational status				
No Formal Education	38(32.2)	80(67.8)	1.82(1.01-3.48)	2.00(1.33-6.75)
Formal Education	18(20.7)	69(79.3)		1
Monthly income				
< 500ETB	8(16.7)	40(83.3)		1
500-999ETB	17(27.9)	44(72.1)	0.52(0.20-1.33)	1.23(0.61-2.49)
> 1000ETB	31(32.3)	65(67.7)	2.38(1.15-5.70)	2.41(1.32-4.63)**
Duration of diagnosis				
< 2 years	19(28.4)	48(71.6)		1
2-5 years	30(33.3)	60(66.7)	3.17(0.38-6.58)	3.81(1.27-6.51)
5-10 years	6(13.6)	38(86.4)	2.51(1.25-6.89)	2.48(1.32-4.64)*
> 10 years	1(25.0)	3(75.0)	2.11(0.12-12.14)	1.19(0.12-12.14)
Hear information about lifestyles				
Yes	39(33.1)	79(66.9)		1
No	17(19.5)	70(80.5)	0.49(0.25-0.95)	0.64(0.29-1.38)
Source of information				
Health professionals	31(35.2)	57(64.8)		1
Different Medias	6(33.3)	12(66.7)	0.91(0.53-12.53)	2.64(0.77-9.09)
Family and friends	3(21.4)	11(78.6)	1.99(0.52-27.72)	4.44(0.81-27.72)
Co-morbidity				
Yes	23(25.8)	66(74.2)	0.87(0.03-0.93)	0.28(0.13-0.61)*
No	33(28.4)	83(71.6)		1

* Significant at p-value < 0.05, ** Significant at p-value < 0.001

## Discussion

This study was conducted with intention to assess lifestyle modification practices and associated factors among hypertensive patients. Control of hypertension represents a major challenge and requires attention to both pharmacological and non-pharmacological treatment. Measurement of the rates of lifestyle practices and medications together with the identification of its determinants is of ultimate importance for the design effective strategies to control hypertension. The study revealed that 92 (44.9%) had good basic knowledge regarding hypertension which is low compared with a study done in Jimma showed 67.7% participants have good knowledge regarding hypertension [29]. however, the finding of a study in Egypt supports with the current finding [30].

Eighty-eight (73.2%) reported their source of information about recommended lifestyle were health professionals. This is supported by finding from study in Nigeria [17].

From the participants (92.2%) were non-smokers and (89.7%) were abstained from alcohol drinking. This is supported by study finding [20]. This could be due to social and cultural practices that discourage alcohol drinking and smoking.

Adequate physical activity has been shown to have many health-promoting effects and has a direct role in reducing blood pressure [31, 32]. In this study very fewer patients 56(27.7%) practiced lifestyle modification. This finding is lower than a study done in china in which (70%) of participants practice lifestyle modification and higher than from study done in Saudi Arabia [21, 33]. This might be due to difference educational back ground of patients and level of awareness about lifestyle modification and its advantages. It also might be due to patients relay only on medication considering lifestyle modification has no effect on their blood pressure.

Among lifestyles modifications only (16.1%) of the participants practice regular exercise 30 min per day for most of the days in a week, which is lower than (89%) and (43.7%) patients physically active in studies done India and Addis Ababa respectively [20, 34]. A possible explanation could be lack of organized setups that are favorable for exercise. Another possible explanation could be poor knowledge on the importance of physical activity in the management of hypertension. A similar study done in Saudi Arabia showed that only (11.1%) of patients practiced physical activity most of the days in a week. Another study was done in the USA in African Americans also showed the majority of the patients practiced physical activity that is higher than this study’s finding [35].

The mean (+SD) practice score of low salt diet was 4.59(±2.03). One hundred eighteen subjects (57.5%) have poor low salt diet practice. This finding was higher than a study done in Saudi [21] but this finding is lower than the finding of a study done in hypertensive patients in Jimma university specialized hospital (Ethiopia) which showed 80% of hypertensive patient avoid salt in their diet. In addition, a study done in the United States showed low salt diet practices among African Americans [35]. This may be due to the socio-cultural practice of the community and poor knowledge about the effect of high salt diet in blood pressure control. In addition, it could be due to the intention of individuals to make the food tastier by adding salt that is common in Africa [29].

The mean (+SD) of weight management practice of study subjects was 29.36(±13.43) with the maximum score of 50. Eighty-six (41.9%) only have good weight management practice. The finding is in consistence with study done in the United States. This finding was also supported by study in Saudi and Nigeria [21, 35, 36].

Higher age (>65 years) was significantly associated with lifestyle modification practice Patients. Age Above 65 years old was 73% less likely to practice lifestyle modification (AOR = 0.27, 95%CI: 0.13-0.61). This is in line with the study done in China and Nigeria [22, 33]. This could be due to older persons have less education, decreased cognitive function and have more co-morbidities which may inhibit the practice of the lifestyles. Another explanation might be younger patients were more likely to be educated, eager to control their blood pressure by practicing the lifestyle modification.

The results of the analysis showed patients without formal education were 2 times more likely to practice lifestyle modification (AOR = 2.00 95%CI: 1.33-6.75) as compared to those who attended formal education. This is inconsistent with the study done Nigeria and Botswana in which as educational status increase practice of lifestyle modification was higher [22, 23].

Average monthly income was significantly associated with lifestyle modification practice. Participants with a monthly income of more than 1000ETB were 2.4 times more likely to practice lifestyle modification (AOR = 2.41, 95% CI: 1.32-4.63). This is supported by the finding from the study in Saudi in which level of monthly income was highly significantly associated with lifestyle modification practice [21]. This could be due to individuals with low income could face to manage their diet properly and could not get favorable setups to do physical exercise. This also might be due to cultural differences that influence the living style of the individuals.

Patients on treatment for 5-10 years were 2.48 times (AOR = 2.48 95%CI: 1.32-4.64) more likely to practice lifestyle modification as compared to those patients who were in treatment for less than 2 years. This finding is supported by different studies that show patients on longer duration of treatment had good lifestyle modification practice [21, 36]. This might be due to continued counseling’ and health education.

Patient with co-morbidity was 72% less likely to practice lifestyle modification practices (AOR = 0.28, 95%CI: 0.13-0.61) compared to patients without co-morbidity. Hypertension with the presence of other co-morbidity is very difficult to control. Co-morbidities can worsen the conditions of the patient and make them unable to adhere to practice lifestyle changes [34]. This finding is consistent with the study done in India and Ethiopia (Addis Ababa) that showed patients without co-morbidity were more likely to practice lifestyle modification [20, 34]. This difference could be due to the difference in living standard and cultural differences. In addition, it might be due to difference educational status. Peoples in an urban area are more educated and might have awareness about their blood pressure.

The main limitation of this study is lack of adequate similar studies in our country, which made comparison difficult for the lifestyle changes. In addition, the data was self-report from the participants; there may be the denial of poor practices from the respondents, which affects the result of the study.

## Conclusion

This study revealed lifestyle modification practice is low among the hypertensive patients. Lifestyle modification through changes in eating patterns, abstaining from alcohol, weight management, smoking cessation and regular physical activity forms part of important and effective treatment strategies for hypertension. Regardless of other indicated treatments, all hypertensive patients who need to control their blood pressure should be given advice and support to achieve and maintain lifestyle practices. Age, Duration of the hypertension diagnosis, educational status, average monthly income, and co-morbidity were factors significantly associated with lifestyle modification practice.

## Abbreviations

AOD: Adjusted odds ratio
CI: Confidence interval
IRB: Institutional Review Board
SPSS: Statistical Package for Social Sciences
